# Supplementary material for: Patterns of Intron Gain and Loss in Fungi
Source: PLoS Biol. 2004 Nov 30;2(12):e422. doi: 10.1371/journal.pbio.0020422 (PMC532390; doi:10.1371/journal.pbio.0020422)
Supplement: Table S1 — Also available at http://genes.mit.edu/NielsenEtAl/. (4.3 MB ZIP). [file pbio.0020422.st001.zip › NielsenEtAl/html/317.html]

AN0233.1.NCU05853.1.MG08446.1.FG05720.1


```
 CLUSTAL W (1.82) Multiple Sequence Alignments - Introns Inserted


Sequence 1: MG08446.1	541 aa
Sequence 2: FG05720.1	537 aa
Sequence 3: AN0233.1	503 aa
Sequence 4: NCU05853.1	541 aa
Alignment Length: 559 aa
Number Identitical Residues: 195 aa
Alignment Score (without introns) 11161


MG08446.1 	MAIIKAAD-----GTADPVLTNLVAQDKVRFWMKPNLRRMYIFLFLCCMGVEMTSGFDSQ
NCU05853.1	---MGGAG--GDFGGSDPVISRLAKEDPIPWYKKPNLRFLYFMLFPTCMGIELTSGFDSQ
FG05720.1 	---MVNVG-----SAADPIVTRLVEEDKTPWYKKPNLRIMYVWLFFCCMGVEMTSGFDSQ
AN0233.1  	---MVAIGKQREEDVSEPILETLIQNDCTPWYKKPNLRNLYLILFPACMGIEITSGFDSQ
          	   :   ... . . ::*::  *  :*   :: ***** :*. **  ***:*:*******

MG08446.1 	LINTMLFTEPYLLY1FGNGWRNKDGKLGIEPNLL~GIMNASYNLGSILGVPLAPWFSHKF
NCU05853.1	MINALQILESWIQY1FDNP----------QGALK~GIISAAYSLGAILSLPLVPIINDRF
FG05720.1 	LINTLQYSQTFHHY1LGDGRMDDKGKYAIEPGLL~GFVNSCYQLGSIFAVPVAPWFAQKY
AN0233.1  	IIN-----------~------------------T0GFLAAAYSLGAVLALPVVPWINQKV
          	:**                                *:: :.*.**:::.:*:.* : .: 

MG08446.1 	GRRWSIMAGSIIMIVGAVIQAFAQHA~AMYIIARIILGIGIVFAIIAGAAMIGELAYPKE
NCU05853.1	GRRWSIALGSIVMIVGAIIQGFSQHV1GMYIVARMILGFGIPTCIVSGSSLIGELAYPKE
FG05720.1 	GRRWSIMLGSLIMVVGAIIQGFAQHV1GMYIVARIILGAGILFCIISGAALIGELGYPKE
AN0233.1  	GRRWTIMFGSCISLVGAILQGFSNGA1AMYCIARIVLGFGIPFCIVAGSSMLGELGYPKE
          	****:*  ** : :***::*.*:: . .** :**::** **  .*::*::::***.****

MG08446.1 	RAMLTSLFNASWFIGAIVASAVAIETVNIKGDWAWRLPSLLQICPSLLQICTVF~FLPES
NCU05853.1	RPVLTSLFNVSYFVGQIVAAAIVFGTNSIASNWGWRIPSLLQICPSLLQLAFVF2FIPES
FG05720.1 	RATLTSLFNSSYFVGQILASAVALGTTSIKTNWAWRIPSLLQICPSLVQIATVF2LLPES
AN0233.1  	RPILTSLFNSSYFIGQITAAAVGLGTVTIPSDWAWRVPSLLQIAPAMVQIVTVM2FLPES
          	*. ****** *:*:* * *:*: : * .*  :*.**:******.*:::*:  *: ::***

MG08446.1 	PRYLISQDRDDEAFEILTKYHAEGDASSPIVQAEMAQIRTTIKLEME-NSKQTWADVLKT
NCU05853.1	PRWLITKDRSQEAHDILKKYHGEMERGEEFVAAEFAQMQAVIRLEYETVSKSSWMDLLRT
FG05720.1 	PRFLISKDRTEEAAAILVKYHAEGDANSPLVQAEIVQIRETIKAEME-ASKQSWLSVIRT
AN0233.1  	PRYLVSKDRTDEAVEILTKYHAEGDRDSVIVKAEIAQIERTILLERE-ESKQSWGDLFRT
          	**:*:::** :**  ** ***.* : .. :* **:.*:. .*  * *  **.:* .:::*

MG08446.1 	AGMRRRFFITIFIGLFTQMSGNTLLS~YYSSLLYDLMGYTETSLKTRLNMADKCWGLINA
NCU05853.1	PGMRKRLLISSMLGLFTQWSGNTLIS~YFLGDLLKMIGFTDSTFIQKINVSIACWSLFCG
FG05720.1 	AGMRRRFLVTVFIGLFTQLSGNTLMS~YYSGKLFELMGYTSDYTKTRINLAFACWSLMNA
AN0233.1  	AGMRRRLFITAFLGLFTQWSGNTLIS2YYLSDLLDMVDIKDSVVKSKINVGIACWGLVCG
          	.***:*:::: ::***** *****:* *: . * .::. ..     ::*:.  **.*. .

MG08446.1 	VALALIVVRFPRRRMYMLSAASMLCVFVAMTVCFYYLRQATNDGVRNYPAGAAALFFMYA
NCU05853.1	VTVSLLVTRIRRRIMYMACTISLLLCYIAWTVSMERAMTGKANGTPNNGANIATLFFIYM
FG05720.1 	TALALIVPRFKRRHMYMLSATLMLLVFIAITVSLQQI-QATPVGQKNNAAGIAALFFYFA
AN0233.1  	TTLALTAPRFKRRTMYLTTACSLLSVYIAWTISMERAMT-----TETKAAAVLTILFIFL
          	.:::* . *: ** **:  :  :*  ::* *:.:            .  *   :::* : 

MG08446.1 	YGPCYNMGNNALTYT~YLVELWPYAQRSRGIGVQQIFGKAGGFFSTYVNPIAINALDWRY
NCU05853.1	YSPCYNMGYNALTYT1YMVEVWPYAERSRGIAVFQLFGRLAGFFTTFVNPIGLKNVGWKY
FG05720.1 	YSPTYNIGNNALTYT1YLVELWPYAMRSRGIGVQQIFGKLAGFFSTNVNSIALDAIKWKY
AN0233.1  	YSPAYNLGYNALTYT1YLIEIFPYFNRSRGLSWFQFYGRGATFFATYVNPIGLDRIEWRW
          	*.* **:* ****** *::*::**  ****:.  *::*: . **:* **.*.:. : *::

MG08446.1 	FAIYCGWIGFEFIFIYLMYPETYGRTLEELTF-~-L1FEGKEYTDQANAAVQKQLESDVT
NCU05853.1	LISYCCWLAFEVCFVYFMFPETMGRTLEELTF-~-M~FEGEDLQRQANAAAEKVVNHTEH
FG05720.1 	LAIYCGWIVFEFLIIYLLYPETSGRTLEELTFH1PV~FEDDELKEKATAAVEKQIHFGDA
AN0233.1  	LIVYCCWLAFEVVFIYFFFPETSGRTLEELSF-~-M1FEGREKADEVAAAVHKQIDETEH
          	:  ** *: **. ::*:::*** *******:*   : **. :   :. **..* :.    

MG08446.1 	A----------LPREK0GSLDHDALILA
NCU05853.1	DTTGLGRRSTNENKLG~GSAEVKEHVP-
FG05720.1 	K----------EEENK~TQVQTREVA--
AN0233.1  	K--------------G~ATTEIREKV--
          	                    :
```
